# Supplementary material for: Maximal expiratory pressure is associated with reinstitution of mechanical ventilation after successful unassisted breathing trials in tracheostomized patients with prolonged mechanical ventilation
Source: PLoS One. 2020 Mar 10;15(3):e0229935. doi: 10.1371/journal.pone.0229935 (PMC7064239; doi:10.1371/journal.pone.0229935)
Supplement: S1 File — Additional information regarding the results of the study. (DOCX) [file pone.0229935.s001.docx]

Supplementary Information

**Maximal expiratory pressure is associated with reinstitution of mechanical ventilation after successful unassisted breathing trials in tracheostomized patients with prolonged mechanical ventilation**

Shwu-Jen Lin^1^, Jih-Shuin Jerng^2^*, Yao-Wen Kuo^1^, Chao-Ling Wu^1^, Shih-Chi Ku^2^, Huey-Dong Wu^1^

**Results**

Of the 454 patients, 290 (63.9%) had valid paired sets of data of weaning parameters, including one set measured before weaning and another set measured after successful weaning before the patients were transferred to the general ward. Table 1S summarizes the comparison of demographic and clinical characteristics of the 290 patients (Groups A) with the 164 patients with weaning parameters only before unassisted breathing trials (Groups B). Those who had paired data measurement general had longer duration of MV use at the ICU (31.9±31.1 days vs. 23.7±15.6 days, *p*=0.003) as well as at the RCC (11.4±8.4 days vs. 7.7±6.8 days, *p*=0.003). More patients in Group A had a history of extubation failure at the ICU (34.5% vs. 19.5%, *p*=0.001). Fewer patients in Group A were successfully weaned by the direct liberation protocol (37.2% vs. 55.5%, *p*<0.001), but upon transfer to general ward, the two groups had similar vital sign status, including heart rate, respiratory rate, mean arterial pressure and temperature (Table 1S).

**Table 1S.** Comparison of clinical and demographic characteristics of the patients in this study according to the availability of paired weaning parameter data sets (Group A) or data only before weaning (Group B)

| Characteristic | Total (n=454) | Group A (n=290) | Group B (n=164) | *p*-value |
| --- | --- | --- | --- | --- |
| Age, mean±SD | 68.9±16.2 | 69.6±15.8 | 67.7±16.9 | 0.335 |
| Sex, male (%) | 299 (65.9%) | 185 (63.8%) | 114 (69.5%) | 0.217 |
| Co-morbidity |  |  |  |  |
| Hypertension | 157 (34.6%) | 95 (32.8%) | 62 (37.8%) | 0.278 |
| Congestive heart failure | 147 (32.4%) | 110 (37.9%) | 37 (22.6%) | 0.001 |
| Diabetes mellitus | 123 (27.1%) | 82 (28.3%) | 41 (25.0%) | 0.451 |
| Neurologic disease | 114 (25.1%) | 83 (28.6%) | 31 (18.9%) | 0.022 |
| Chronic kidney disease | 113 (24.9%) | 80 (27.6%) | 33 (20.1%) | 0.077 |
| Malignancy | 112 (24.7%) | 72 (24.8%) | 40 (24.4%) | 0.917 |
| Liver cirrhosis | 46 (10.1%) | 30 (10.3%) | 16 (9.8%) | 0.842 |
| COPD | 36 (7.9%) | 27 (9.3%) | 9 (5.5%) | 0.148 |
| Main conditions related to MV use |  |  |  |  |
| Pneumonia | 251 (55.3%) | 161 (55.5%) | 90 (54.9%) | 0.895 |
| Post-operation MV use | 148 (32.6%) | 88 (30.3%) | 60 (36.6%) | 0.173 |
| Cerebral hemorrhage/head injury | 144 (31.7%) | 79 (27.2%) | 65 (39.6%) | 0.006 |
| Sepsis | 72 (15.9%) | 53 (18.3%) | 19 (11.6%) | 0.061 |
| Heart failure | 48 (10.6%) | 36 (12.4%) | 12 (7.3%) | 0.090 |
| ARDS | 20 (4.4%) | 15 (5.2%) | 5 (3.0%) | 0.348 |
| Spinal cord injury | 12 (2.6%) | 7 (2.4%) | 5 (3.0%) | 0.763 |
| Admitted from medical ICU | 242 (53.3%) | 163 (56.2%) | 79 (48.2%) | 0.099 |
| Intervention at the ICU |  |  |  |  |
| Tracheostomy | 444 (97.8%) | 290 (100.0%) | 154 (93.9%) | <0.001 |
| Renal replacement therapy | 88 (19.4%) | 57 (19.7%) | 31 (18.9%) | 0.845 |
| Inhaled nitric oxide | 10 (2.2%) | 8 (2.8%) | 2 (1.2%) | 0.283 |
| Prone positioning | 7 (1.5%) | 4 (1.4%) | 3 (1.8%) | 0.709 |
| ECMO | 6 (1.3%) | 5 (1.7%) | 1 (0.6%) | 0.318 |
| Extubation failure before tracheostomy | 132 (29.1%) | 100 (34.5%) | 32 (19.5%) | 0.001 |
| Extubation times before tracheostomy | 1.3±0.5 | 1.4±0.6 | 1.2±0.5 | <0.001 |
| ICU days before RCC transfer | 25.7±17.0 | 27.1±17.8 | 23.4±15.4 | 0.227 |
| RCC length of stay | 15.5±8.1 | 16.9±8.3 | 12.9±6.9 | 0.003 |
| Total MV days | 38.7±27.9 | 42.8±31.8 | 31.4±16.7 | <0.001 |
| MV days at ICU | 28.6±26.8 | 31.9±31.1 | 23.7±15.6 | 0.003 |
| MV days at RCC | 10.1±8.1 | 11.4±8.4 | 7.7±6.8 | <0.001 |
| APACHE II score on admission to ICU |  |  |  |  |
| APACHE II score on admission to RCC | 14.9±5.0 | 15.1±4.9 | 14.6±5.2 | 0.479 |
| APACHE II score at RCC discharge |  |  |  |  |
| Heart rate at RCC discharge | 87.0±14.5 | 87.4±14.8 | 86.3±13.9 | 0.464 |
| Respiratory rate at RCC discharge | 20.7±4.7 | 21.0±5.0 | 20.2±4.1 | 0.084 |
| Mean blood pressure at RCC discharge | 88.1±12.4 | 87.5±12.0 | 89.7±13.1 | 0.063 |
| Fever (BT>38.3 degree) at RCC discharge | 2 (0.4%) | 2 (0.7%) | 0 (0%) | 0.537 |
| Mode of weaning success |  |  |  |  |
| Direct liberation | 199 (43.8%) | 108 (37.2%) | 91 (55.5%) | <0.001 |
| Stepwise protocol | 245 (54.0%) | 175 (60.3%) | 70 (42.7%) |  |
| Slow weaning | 10 (2.2%) | 7 (2.4%) | 3 (1.8%) |  |
| In-hospital mortality | 47 (10.4%) | 31 (10.7%) | 16 (9.8%) | 0.754 |

APACHE II: acute physiology and chronic health evaluation II; ARDS: acute respiratory distress syndrome; BT: body temperature (Celsius); COPD: chronic obstructive pulmonary disease; ECMO: extracorporeal membrane oxygenation; ICU: intensive care unit; MV: mechanical ventilation; RCC: respiratory care center.

Table 2S showed the comparison of weaning parameter data before unassisted breathing trial and after successful weaning of the 290 patients with paired *t*-test. There was no significant evolutional change in the data (Table 2S).

**Table 2S.** Comparisons of weaning parameters before unassisted breathing trial and after successful weaning in 290 patients.

| Weaning parameter | Before unassisted breathing trial | After successful weaning | p-value |
| --- | --- | --- | --- |
| P_I_max | 37.5±12.8 | 36.8±12.3 | 0.347 |
| P_E_max | 40.0±16.7 | 38.4±16.5 | 0.136 |
| V_T_ | 338.7±123.1 | 335.6±122.0 | 0.665 |
| V_E_ | 8.3±2.9 | 8.1±2.8 | 0.280 |
| RR | 25.5±7.1 | 24.9±6.3 | 0.235 |
| RSBI | 87.8±46.9 | 85.4±43.0 | 0.386 |

P_I_max: maximal inspiratory pressure; P_E_max: maximal expiratory pressure; V_T_: tidal volume; V_E_: minute ventilation volume; RR: respiratory rate; RSBI: rapid shallow breathing index
