# Supplementary material for: Maximal expiratory pressure is associated with reinstitution of mechanical ventilation after successful unassisted breathing trials in tracheostomized patients with prolonged mechanical ventilation
Source: PLoS One. 2020 Mar 10;15(3):e0229935. doi: 10.1371/journal.pone.0229935 (PMC7064239; doi:10.1371/journal.pone.0229935)
Supplement: Attachment — Submitted filename: Responses to Reviewer Comments.docx [file pone.0229935.s003.docx]

**Responses to Reviewer 1 Comments**

Major comments:
1. In Abstract, the statement of “…similar PImax, tidal volume, minute ventilation, respiratory rate, and rapid shallow breathing index before unassisted breathing trial with those without reinstitution.” need to be reedited to “compared with those without reinstitution”.

**Response**: We have revised this sentence by adding ‘compared’ to it as suggested. Please refer to Abstract of the revised manuscript for the change.

2. In abstract, the authors reported the results of Cox regression analysis for detecting the factors associated with MV reinstitution within 60 days in hospital. There are two criteria (MV reinstitution before discharge and within 60 days in hospital) to categorize the study patients into the study and control group. Which one is the end-point for Cox regression analysis?

**Response**: To avoid confusion, we have revised the manuscript to focus on “in-hospital MV reinstitution within 60 days” (referred as ‘reinstitution’) as the primary end-point of this study. Therefore, all of the results were rearranged for this end-point. For Cox regression, we only show the result related to this “in-hospital MV reinstitution within 60 days” end-point. Please refer to page 9 of Methods, Table 3 and Results sections the revised manuscript for the changes.

3. In conclusion, the authors declare that “Interventions to enhance expiratory strength in tracheostomized patients are warranted.”. However, in the current study, the study design is retrospective observational and there is no intervention to prove this concept.

**Response**: We have revised this section and replaced the original sentence with ‘Further studies are needed to confirm the relevance of expiratory muscle strength and cough function with regards to patient outcomes after they have been successfully weaned based on the operational criteria.’ Please refer to Conclusions on Page 21 of the revised manuscript for the change.

4. In Data Collection, the timing for weaning parameters measurement is not clear and consistent during daily protocolized weaning process. Is it measured for (1). initiating unassisted breathing for 12 hours for 2 consecutive days during screening period, (2).direct liberation trial with UBT for 5 days, (3). stepwise liberation, (4). every 14 days during the RCC stay, (5). before patients transferred to ordinary ward. If patients had several measurements of weaning parameter for different purpose, which one is selected to be analyzed?

**Response**: The timing for weaning parameter measurement was always before the initiation of unassisted breathing trial (UBT). We have added a description to explain that in this study, as the timing of initiating UBT was determined by the clinicians not necessarily linked to the measurement of weaning parameters, we only selected the data of weaning parameters most close to the beginning of UBT. We have provided the information in the description of the Results section that the measurement of parameters was performed on 9.7±7.9 days before the first day of continuous UBT. Please refer to page 8 of Methods section and page 13 of Results section for the change.

5. In Materials and Methods, there is no primary outcome measurement mentioned. There is no study design for how to group patients. If the study patient is expired, how to define the group for the case?

**Response**: As we have above responded, we have revised the manuscript to focus on “in-hospital MV reinstitution within 60 days” as the primary end-point of this study. A patient was allocated to the “non-reinstitution” group if the patient died in the hospital without reinstitution of MV. Please refer to page 9 of Methods, Table 3 and Results sections the revised manuscript for the changes.

6. In Results, the authors reported that the median interval for reinstitution of MV was 8.0days, and 43.2% reinstitutions occurred in <7days after transfer to the general ward. The mean length of stay in RCC was 15.5days, and the authors investigate the reinstitution of MV after RCC discharge. The conditions on RCC discharge have a critical determinant on MV reinstitution in ordinary ward. However, only ICU conditions are showed on Table 1 and analyzed for reinstitution of MV in ordinary ward.

**Response**: We have retrieved relevant physiologic data for the calculation of APACHE II scoring for patients on the day they were transferred out of the RCC. The calculated data for APACHE II at RCC discharge as well as vital signs are added to Table 1. With the multivariable Cox regression, we have included the APACHE II scores on ICU admission, on RCC admission and at RCC discharge for the analysis, and the results, as shown in Table 3, did not show a significant association with the risk of MV reinstitution. Please refer to Table 1 and page 15 in Results for the changes.

7. In Results, the authors reported Table 1S for 290 cases with weaning parameters measured after successful weaning upon transfer out to a general ward. What is the reason to do it in clinical practice?

**Response**: The real-world practice in our institution might include the measurement of weaning parameters for tracheostomized before they are transferred out the RCC after days of un-interrupted UBT, in case there is a concern for reconnection to MV at the general ward; however, the decision for measuring was based on the judgement of clinicians and respiratory therapist, who might provide expert opinions about further care at the general wards. We have added this description to the Methods section. There was no significant evolutional change in the data for each parameter of these 290 patients (Table 2S); therefore, we decided to include only weaning parameters before the unassisted breathing trial for consideration of multivariable Cox regression analysis. Please refer page 9 in the Methods section for the change.

8. In Table 3, the method 1 and method 2 are performed for MV reinstitution before discharge and 60 days before discharge. If the study case is connected to MV after RCC discharge, but liberation from MV before hospital discharge. How to define this patient? This classification in Table 3 (within 60 days) for the study patients is different from that in Table 1 (Reinstitution and non-reinstitution before discharge).

**Response**: The outcomes regarding MV reinstitution at the general ward were described in the Results on pages 10-11. For better display of the information, we have rearranged the Results and Tables. We have revised Tables 1 and 2 to show the comparison of patients with and without in-hospital MV reinstitution within 60 days, whereas the patients who had reinstitution of MV were allocated to the ‘reinstitution’ group. In Table 3, we now only show the results for Cox regression using the time-dependent outcome of “in-hospital reinstitution within 60 days” to avoid confusion to the readers. Please refer to pages 10-11, Tables 1, 2 and 3 for the changes.

9. In table 1, the MV days before RCC transfer is not presented.

**Response**: To Table 1 we have added the data relevant to MV use, including total days of MV use, ICU MV days, and RCC MV days. These data were similar between the reinstitution and non-reinstitution groups of analyses. Please refer to Table 1 in the Results section for the change.

10. In Discussion, paragraph 3, the authors mentioned the factors associated with unsuccessful weaning. However, in the current study, the targeted study population is the ones with the reinstitution of mechanical ventilation after successful unassisted breathing trials.

**Response**: As there was limit reports in the literature regarding the factors associated with MV reinstitution in tracheostomized patients with prolonged MV, we have shortened this paragraph to avoid confusion to the readers. Please refer to the Discussion section of the revised manuscript for the change.

11. In Discussion, paragraph 4, the authors mentioned that “although the reference value for PEmax has not been as frequently discussed in the literature as PImax “. In this study, the values of P_E_max in non-reinstitution and reinstitution group are 43.5cmH2O and 37.4 cmH2O (p = 0.001). Is the 6 cmH2O difference of PEmax clinically meaningful?

**Response**: As this study was conducted retrospectively, we recognized that the patient population in this study was heterogenous that multiple factors affected the outcomes. The data in Results and Table 3 support that factors other than P_E_max affected the outcome independently, such as co-morbidities, complications at the ICU, severity of organ dysfunction at the ICU, and MV duration. Therefore, the univariate comparison of P_E_max between these two groups (reinstitution vs non-reinstitution) might not infer the independent significance of association, or preclude this factor for multivariable analysis.

12. In Discussion, the paragraph 5 and 6 are redundant, Please reedit the content of discussion to focus on the main findings (factors associated with reinstitution of MV) in the current study compared to previous literature.

**Response**: We have reduced the content of these paragraphs to make the Discussion more concise. Please refer to Discussion of the revised manuscript for the changes.

13. In figure 2, it seems rapid reinstitution of MV after RCC discharge in Pemax <=30cmH2O compared to Pemax >30cmH2O. why is the cut-off value of 30cmH2O is selected and how to explain it?

**Response**: There were limited reports in the literature regarding the reference P_E_max value for predicting outcomes for tracheostomized patients. For clinical practice, we also chose to test the discriminating power by the nearest ten value; therefore, we have also performed Cox regression analysis to use P_E_max > 20 cmH_2_O to replace P_E_max > 30 cmH_2_O as the cut-off value, and found that the protective significance for MV reinstitution was lost; therefore, we chose P_E_max > 30 cmH_2_O as the cut-off value. We also addressed this point in the ‘Statistical analysis’ subsection of the ‘Methods’ section.

**Responses to Reviewer 2 Comments**

Major comments.
1) Abstracts. Check the instructions for authors. I don’t think it is relevant to describe the univariate analysis results in this section. Please provide data about outcome, which are missing.

**Response**: We have revised the Abstract section to remove the univariate analysis and add outcome descriptions. Please refer to the revised manuscript for the change.

2) Statistical analysis. Cox Model. Can you add on which parameters you adjusted the model, and how these factors were selected? Since there is an important imbalance between groups, this aspect is paramount.

**Response**: As we had found that the patient population was very heterogeneous, therefore we selected the variables with a *p*-value of less than 0.1 into the multivariable Cox regression. As we have re-performed the Cox regression analysis, the final model now contains 13 variables, with basically P_E_max > 30 cmH_2_O remaining as significant protecting factor for MV reinstitution. Please refer to the revised manuscript at Table 3. We also addressed how to select these factors in the ‘Statistical analysis’ subsection of the ‘Methods’ section.

3) Can you provide SAPS II or APACHE at ICU admission? If baseline severity between groups is different, this point could explain in itself the difference in the outcomes between groups.

**Response**: We have added the results of APACHE II to Table 1, which now contains APACHE II scores on ICU admission, on admission to RCC and at RCC discharge, and the comparisons between the reinstitution and non-reinstitution groups. Please refer to the Results and Table 1 in the revised manuscript. Although the reinstitution group had higher APACHE II scores on ICU admission, on admission to RCC and at RCC discharge scores in univariable analysis, the multivariable Cox analysis showed that none of them were independent factors of risk of reinstitution of MV.

4) Methodology. Can the authors describe their selection of patients with paired sets of data? I don’t exactly understand what is meant by “paired data sets”? This issue appears only in the results and is not described elsewhere. There is a potential selection bias that needs to be clearly addressed in the methodology: there are less cardiac co-morbidities and neurologic insults in Group B. This issue must be further explained and detailed in the statistical analysis section: how did the authors cope with such issue?

**Response**: The real-world practice in our institution might include the measurement of weaning parameters for tracheostomized before they are transferred out the RCC after days of un-interrupted UBT, in case there is a concern for reconnection to MV at the general ward; however, the decision for measuring was based on the judgement of clinicians and respiratory therapist, who might provide expert opinions about further care at the general wards. We have added this description to the Methods section. As further statistical analysis did not show new findings, we decided to remove the statement of statistical analysis for the weaning parameter measurement at RCC discharge. Please refer page 9 in the Methods section for the change.

5) Please provide OR, confidence of Interval and p value for multivariable analysis

**Response**: As in Table 3 the 95% CIs and p values have been provided, we have added hazard ratio (HR), 95%CI and p values to the Abstract for Cox regression. Please refer to the Abstract of revised manuscript for the change.

6) Authors must describe the variable selection process regarding logistic regression and Cox model. I believe that a methodologist should help in the process

**Response**: As has been provided in the response to Comment #2, we have provided the description of variable selection based on our initial findings. Please refer to our response to Comment #2 and the ‘Statistical analysis’ subsection of the ‘Methods’ section. To avoid confuse the readers, we decided to obtain the result of Cox model and remove the logistic regression version. We did actually consult a senior statistician and we have also acknowledged for his help in the ‘Acknowledgments’ section of the revised manuscript

7) The MV duration > 30 days as a protective factor of weaning failure is counter-intuitive and not in line with previous data (Beduneau AJRCCM, Funk ERJ) showing that the duration of MV is a risk factor of weaning failure. Can the authors attempt to explain this finding?

**Response**: We agree that previous reports, mainly focusing on overall weaning prognosis assessment, concluded that prolonged MV > 30 days as a negative predictor for successful weaning. However, as the revised Cox regression analysis did not show MV > 30 days as a significant factor, we decide not to include this factor in the discussion. Please refer to Table 3 of the revised manuscript.

8) I am unsure that the logistic regression model obeys the parsimonious rule (ie 1 variable in the model per 5-10 events maximum). The same question arises for the Cox model. Again, a statistical reviewing seems mandatory. Moreover, these models seem redundant (same risk factors)? What is the new information provided by the Cox model? Did the authors study the same outcome?

**Response**: As we have rearranged the results to focus on 60-day in-hospital MV reinstitution, we have reduced the descriptions of multivariable analysis to only Cox regression analysis. The process of choosing variables is provided in the ‘Statistical analysis’ subsection of the ‘Methods’ section.

A total of 13 candidate predictors were introduced into the multivariable Cox regression and we had 116 events (reinstitution of MV within 60 days), therefore the final presentation in Table 3 would conform to the parsimonious rule. Please refer to the Methods section and Table 3 of the revised manuscript for the changes. In addition, as mentioned at comment#7, we did actually consult a senior statistician and we have also acknowledged for his help in the ‘Acknowledgments’ section of the revised manuscript

9) In the end of the results section, the authors provide new data in a 290 patients’ sample with paired data. What is the difference with the previous sample? These data must be clearly expressed in the Statistical analysis section.

**Response**: We have added descriptions regarding the methods for assessing the paired data mentioned in the Supplement materials. There was no significant evolutional change in the data for each parameter of these 290 patients (Table 2S); therefore, we decided to include only weaning parameters before the unassisted breathing trial for consideration of multivariable regression analysis. Please refer to the Methods sections of the revised manuscript for the changes.

10) An effort should be made, in order to reorganize the results section
**Response**: We have reorganized the Results section for better readability. Please refer to the Results sections of the revised manuscript for the changes.

Minor comments.
1) I believe an English editing, by a native English speaker is mandatory

**Response**: The manuscript had actually been edited by a native English-speaker. We have repeated this English editing process for the revised manuscript.

2) The introduction should be more straightforward. The discussion section must be shortened.

**Response**: We have thereby revised these sections to provide more readability. Please refer to the Introduction and Discussion sections of the revised manuscript for the changes.
